# Long-Term Retrospective Analysis of Parvovirus B19 Infections in Blood Donors (2012–2024): Significant Increase in Prevalence Following the SARS-CoV-2 Pandemic

**DOI:** 10.3390/diagnostics15182313

**Published:** 2025-09-11

**Authors:** Michaela Oeller, Orkan Kartal, Iuliia Trifonova, Nina Held, Alexandra Domnica Hoeggerl, Heidrun Neureiter, Wanda Lauth, Christoph Grabmer, Eva Rohde, Sandra Laner-Plamberger

**Affiliations:** 1 Department of Transfusion Medicine, University Hospital of Salzburg (SALK), Paracelsus Medical University (PMU) Salzburg, Müllner-Hauptstraße 48, 5020 Salzburg, Austria; m.oeller@salk.at (M.O.); o.kartal@salk.at (O.K.); n.held@salk.at (N.H.); a.hoeggerl@salk.at (A.D.H.); h.neureiter@salk.at (H.N.); c.grabmer@salk.at (C.G.); e.rohde@salk.at (E.R.); 2 Team Biostatistics and Big Medical Data, IDA Lab Salzburg, PMU Salzburg, Strubergasse 21, 5020 Salzburg, Austria; iuliia.trifonova@pmu.ac.at (I.T.); wanda.lauth@pmu.ac.at (W.L.); 3 Research Programme Biomedical Data Science, PMU Salzburg, Strubergasse 21, 5020 Salzburg, Austria; 4 GMP Laboratory, PMU Salzburg, Strubergasse 21, 5020 Salzburg, Austria

**Keywords:** parvovirus B19, blood donation, prevalence, SARS-CoV-2 pandemic

## Abstract

**Background/Objectives**: Parvovirus B19 (B19V) is a non-enveloped single-stranded DNA virus transmissible by blood transfusion, with potentially severe outcomes in immunocompromised and pregnant recipients. In this study, we investigated the B19V prevalence in 441,084 blood donations from Salzburg, Austria, collected between 2012 and 2024, focusing on changes in epidemiological dynamics before, during, and after the SARS-CoV-2 pandemic. Additionally, the B19VB19V persistence and its implications for deferral policies were assessed. **Methods**: Donor samples were screened for B19VB19V DNA by qPCR (2012–2024) and for SARS-CoV-2 total anti-N antibodies (2020–2024). B19VB19V prevalence rates, cycle threshold (Ct) values, and seasonal distribution were compared between pre-pandemic, pandemic, and post-pandemic phases. Follow-up testing of initially B19VB19V-positive donors was performed after a 2-year deferral period. **Results**: The B19VB19V positivity rate of 0.13% (2012–2019) significantly decreased to 0.02% during the SARS-CoV-2 pandemic (2020–2022). A substantial increase occurred post-pandemic, with prevalence reaching 1.47% in 2024. Significant lower Ct values were observed in the post-pandemic phase, indicating higher viral loads. Additionally, younger donors (aged 18–45 years) showed significantly lower Ct values. After a 2-year deferral, 39% of re-tested donors remained B19VB19V DNA-positive. **Conclusions**: B19VB19V circulation increased substantially after the SARS-CoV-2 pandemic. Our observation is consistent with international reports and is likely due to an ‘immunity debt’ that has been accumulated due to pandemic-related public health interventions. Targeted B19VB19V screening and strict deferral strategies may be warranted particularly during outbreak periods to protect high-risk transfusion recipients.

## 1. Introduction

Parvovirus B19 (B19V), a non-enveloped single-stranded DNA virus from the *Parvoviridae* family, is the causative agent of erythema infectiosum, often called fifth disease. With a diameter of 20–24 nm, it is one of the smallest known viruses. The B19V genome has a size of 5596 nucleotides, whereby the sequence variability is low. To date, three genotypes are known (1a, 1b, 2, 3a, and 3b), all of which are distributed worldwide [1,2]. In most cases, transmission occurs via respiratory droplets, transplacental transmission or transfusion of blood products [1,2,3]. The incubation period is 4 to 14 days on average. B19V multiplies exclusively in the erythroblasts, the precursor cells of the erythrocytes in the bone marrow [1,4]. The infection initially leads to a temporary suppression of erythropoiesis and thus anemia, which is usually mild and asymptomatic [1]. In immunocompromised or anemic patients, however, this can lead to complications such as aplastic crisis, severe chronic anemia, idiopathic thrombocytopenic purpura, neutropenia, neurologic and rheumatic disease, and chronic fatigue syndrome [5]. In children, B19V usually causes mild symptoms beginning with a blotchy rash on the cheeks, which spreads to the extremities. In healthy adults, the infection is usually asymptomatic, but can also cause various clinical symptoms, with 30–40% of symptomatic cases first showing fever and joint pain before the typical garland-shaped exanthema appears on the trunk or extremities. There is also often reddening of the face. Other rather rare symptoms include the papular purpuric glove-and-sock syndrome, joint pain with swelling (non-erosive arthritis) or a transient aplastic crisis. Although most adults are immune for life after a B19V infection, and around 70% of adults in Europe are seropositive, a new infection can lead to serious complications for pregnant women, such as miscarriage or severe fetal anemia with extensive edema (hydrops fetalis). The risk of miscarriage is around 2–6%, with the greatest risk during the first half of pregnancy [6,7]. After primary infection, lifelong persistence of the virus in various tissues (including skin, synovia, tonsils, liver and serum) is possible [8]. A study from Argentina demonstrated that about 1% of blood donors show such a B19V persistence [9]. Another study indicated a substantially higher B19V persistence rate, as B19V was detected in 22% of skin biopsy samples [10].

If necessary, treatment is usually symptomatic. In immunocompromised patients, immunoglobulins are administered to shorten viremia and stimulate erythropoiesis [11]. Furthermore, blood transfusions are used in severely anemic patients, including unborn children [2]. Even though there are only a few reports of transmission of infections during blood transfusions, transmission of B19V via this route cannot be ruled out [3,12]. The risk of infection through blood and plasma-derived products was clearly correlated to the level of viremia [13]. Therefore, screening blood donations for B19V is performed routinely by many blood banks, e.g., in France, Germany, Austria, and Japan [14,15,16], to ensure blood product safety for transfusion-dependent patients.

The aim of the present study was to determine the B19V prevalence among Austrian blood donors over the last 12 years by retrospective analysis of qPCR test results, including a comparison of the B19V prevalence before, during and after the SARS-CoV-2 pandemic. The analysis of blood donations represents a valuable opportunity to collect epidemiological data for B19V in the adult, per se healthy Austrian population aged between 18 and 70. It further allows investigating B19V persistence in the blood donor cohort. Since data on this issue are rare, donor deferral modalities are handled inconsistently by blood banks, ranging from no deferral at all to a deferral for 2 years. This retrospective data analysis is thus also intended to support the development of guidelines on evidence-based deferral modalities for the blood donors concerned.

## 2. Materials and Methods

### 2.1. Ethical Statement

For the present study, human EDTA plasma and serum utilized for routine laboratory diagnostics as part of standard blood donation processing according to European and local regulations were used [17,18,19]. All blood donors, who gave signed informed consent on the screening for infectious disease parameters, and written signed consent on the use of leftover material and the data analysis for research purposes, were included in the present study. The ethical committee of the Paracelsus Medical University Salzburg approved the study (ethical vote number PMU-EK-2025-0001). The work described was carried out in accordance with the 1964 Helsinki Declaration and its later amendments or comparable ethical standards. Samples were processed anonymously to protect the privacy of each donor.

### 2.2. Sample Collection, Study Design and Cohort Characteristics

For the present retrospective study, 441,084 voluntary blood donations collected throughout the Federal State of Salzburg between 2012 and 2024 were included. The cohort characteristics of blood donors are summarized in Table 1. As described previously, the distribution regarding sex, ABO blood group and age is typical for Austrian blood donors [20]. For data analysis, we have defined comparable periods as pre-pandemic (2017–2019), pandemic (2020–2022), and post-pandemic (2023–2024). The cohort characteristics for these subgroups are comparable to the whole group of blood donors (Table 1).

**Figure 1:**
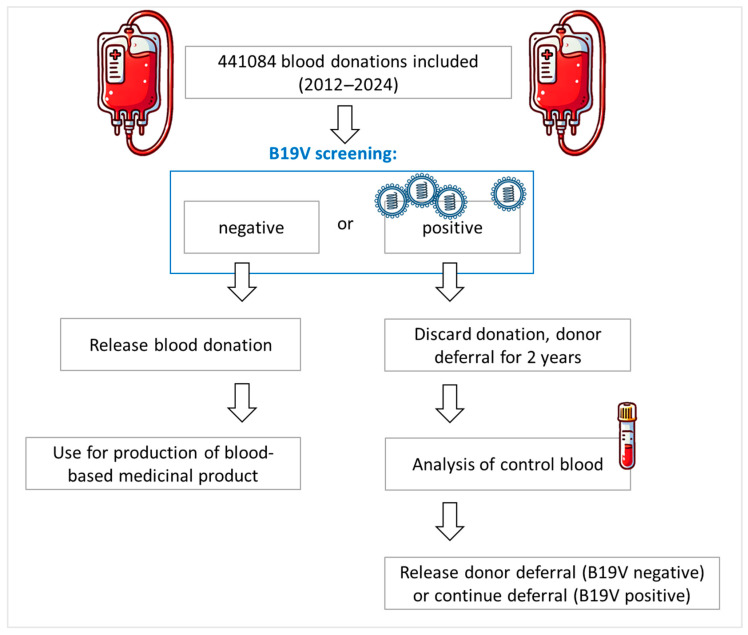
Donor deferral modalities in case of a positive B19V blood donation at the Department of Transfusion Medicine, University Hospital of Salzburg.

All blood donor samples were screened at the Department of Transfusion Medicine, University Hospital of Salzburg for the following infectious disease parameters as part of the standard work-up of each blood donation: HIV, HBV, HCV, HAV, B19V, Syphilis, and SARS-CoV-2. EDTA plasma samples were applied for molecular biological screenings, including screening for B19V, while serum samples were used for the serological screening process including SARS-CoV-2 total anti-N antibodies. Donations that screened negative for B19V were subjected to the production of blood-based medicinal products, while B19V-positive donations were discarded and the donor was deferred from further donations for 2 years. This 2-year deferral was applied to avoid multiple discarding of blood donations of the donor affected. After the deferral, a B19V PCR screening of a control blood sample was screened for B19V. If still positive for B19V DNA, the donor was deferred for another two years, while a negative result led to a release of the deferral. The B19V deferral modalities are summarized schematically in Figure 1.

### 2.3. Nucleic Acid Testing Based on Real-Time Polymerase Chain Reaction (qPCR)

For the detection and quantitative determination of B19V DNA in EDTA plasma from blood donors, the cobas DPX nucleic acid amplification test (Roche Diagnostics, Basel, Switzerland) was applied using a fully automated cobas s 201 system (2012–2014) or a cobas 6800 molecular analyzer system (2015–2024) (both Roche Diagnostics) as described in the manufacturer’s instructions. According to the manufacturer, the linearity range of the DPX B19V quantification is between 40–1 × 10^9^ IU/mL. The DPX assay detects B19V genotypes 1, 2, 3a, and 3b. In brief, the EDTA plasma samples were pooled using a Hamilton Microlab STAR robot (Hamilton Company, Reno, NV, USA; for cobas s 201 system) or a cobas p 680 robot (for cobas 6800 system, both Roche Diagnostics, Basel, Switzerland) with up to 96 donations per minipool. DNA of pooled samples was extracted and amplified together with a DNA quantification standard applying TaqMan Polymerase and specific TaqMan probes provided with the DPX assay kit. The use of the quantification standard in combination with three external controls (a high-titer positive control, a low-titer positive control, and a negative control) allowed the quantitative determination of the B19V viral load. The cobas devices were set to consider a titer of 1 IU/mL as positive for B19V and positive minipools were automatically forced to single resolution to identify the B19V-positive donation(s). Any reactive donation was excluded from further processing. Results are shown as cycle-threshold (Ct) values, viral load is given in IU/mL. Ct and titer values presented in the present study are results from single measurements for each individual donation.

### 2.4. Serological Screening for SARS-CoV-2 Total Anti-N Antibodies

As described previously [20,21,22], the semi-quantitative Elecsys Anti-SARS-CoV-2 (ACOV2) total antibody electrochemiluminiscence immunoassay (ECLIA, Roche Diagnostics, Basel, Switzerland) was used to screen for SARS-CoV-2 anti-N total antibody (including IgM, IgG and IgA). This type of antibody is produced after a SARS-CoV-2 infection and remains largely unaffected by vaccinations. According to the manufacturer, this screening assay, which was conducted using a cobas8000-e801 device (Roche Diagnostics, Basel, Switzerland), detects but does not discriminate all SARS-CoV-2 variants known so far.

### 2.5. Statistical Analysis

Ct values were selected as the primary quantitative measure for the analysis. Although titer values were also recorded, some were reported in a non-numeric form as ‘B19 < Titer min’ or ‘B19 > Titer max’. Therefore, the titer values were classified using 1.1 × 10^9^ IU/mL (T1) as the upper cut-off and 39 IU/mL (T2) as the lower cut-off, based on the minimum and maximum detectable titers specified by the manufacturer. Values falling between these limits were divided into subgroups using the 75th percentile of all titer values as the threshold (T2 and T3 respectively). The Kruskal–Wallis test was used for group comparisons between two or more groups (e.g., age groups, blood types, pandemic phases), while the Wilcoxon rank-sum test was used for comparisons between two independent groups (e.g., sex). Following statistically significant Kruskal–Wallis results, post hoc tests were conducted using Dunn’s test, for which Bonferroni was used to account for multiplicity. Statistically significant results are marked with asterisks as indicated. The significance threshold was set at α = 0.05. Boxplots were used to visualize the data and to show the Ct or titer distributions by group, while stacked bar plots were generated to display the titer groups per year. All analyses were performed using R [23], version 4.4.0.

## 3. Results

### 3.1. Epidemiologic Dynamics of Parvovirus B19 Between 2012 and 2024

**Figure 2:**
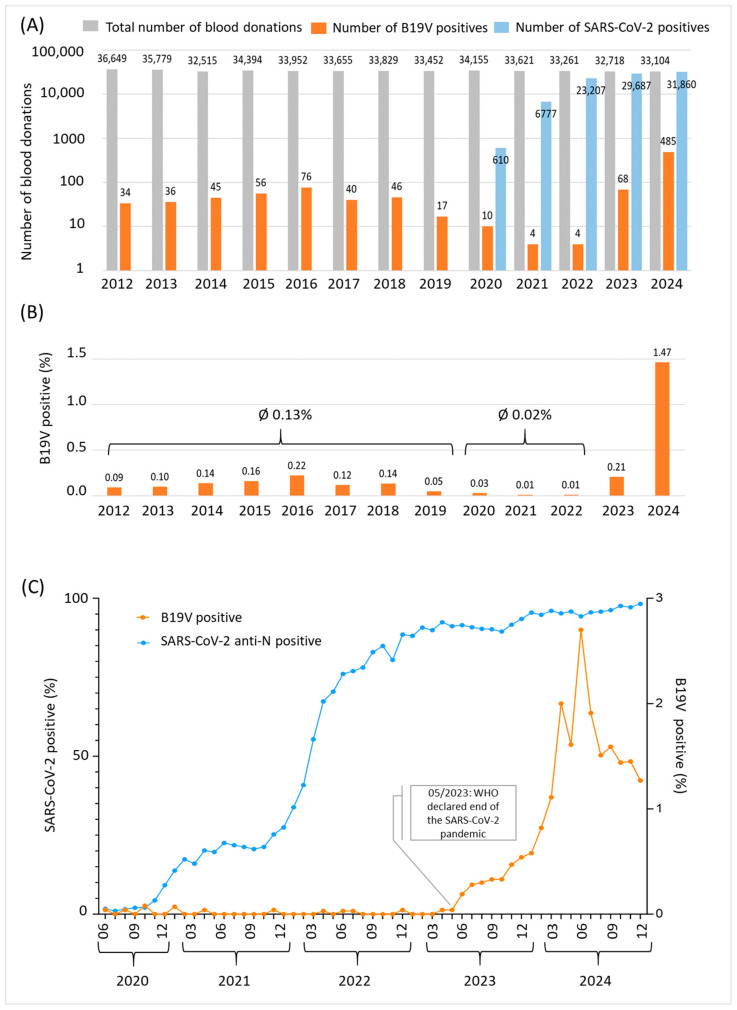
B19V epidemic dynamics in Salzburg, Austria between 2012 and 2024. (**A**) Data shown are total numbers of blood donations (grey), numbers of parvovirus B19 positive blood donations (orange) and numbers of SARS-CoV-2 total anti-N positive donations (blue) per year. A logarithmic scale was chosen to increase visibility of B19V-positive donations. In sum, 441,084 blood donor samples were included. (**B**) B19V prevalence rate per year between 2012 and 2024. (**C**) SARS-CoV-2 (blue, left y-axis) and B19V (orange, right y-axis) prevalence rates among blood donors given in percent between June 2020 and December 2024. The rate of B19V positivity increased significantly after the WHO declared an end to the public health emergency of international concern in May 2023.

The Department for Transfusion Medicine of Salzburg screened 441,084 blood donor samples between 2012 and 2024 for B19V (average: 33,900 per year). Of these, 921 were screened positive for B19V (overall positivity rate: 0.21%). The total numbers of screened voluntary blood donations and detected B19V-positive samples for each year are shown in Figure 2A. Between 2012 and 2019, 44 samples were screened positive for B19V on average each year, which is equivalent to a positivity rate of 0.13% (Figure 2B). During the SARS-CoV-2 pandemic (2020–2022), the B19V prevalence rate significantly decreased to 0.02%, while the number of SARS-CoV-2 positive donations sharply increased (Figure 2A,C). On 5 May 2023, the World Health Organization (WHO) declared the end of the public health emergency of international concern (https://www.who.int/news/item/05-05-2023-statement-on-the-fifteenth-meeting-of-the-international-health-regulations-(2005)-emergency-committee-regarding-the-coronavirus-disease-(COVID-19)-pandemic, accessed on 15 June 2025). Countries worldwide, including Austria, followed the WHO’s recommendation and discontinued SARS-CoV-2 mitigation strategies such as lockdowns, mask-wearing and social distancing. As shown in Figure 2C, there was a strong increase in parvovirus B19 infections detected in blood donors exactly from this point onwards. The peak of B19V infections was reached in June 2024 with a prevalence rate of 2.7% (Figure 2B). Subsequently, the number of B19V-positive blood donors decreased rapidly, reaching 1.27% at the end of December 2024 and 0.5% in March 2025 (data not shown).

### 3.2. Significantly Increased B19V Viral Titers After the SARS-CoV-2 Pandemic

**Figure 3:**
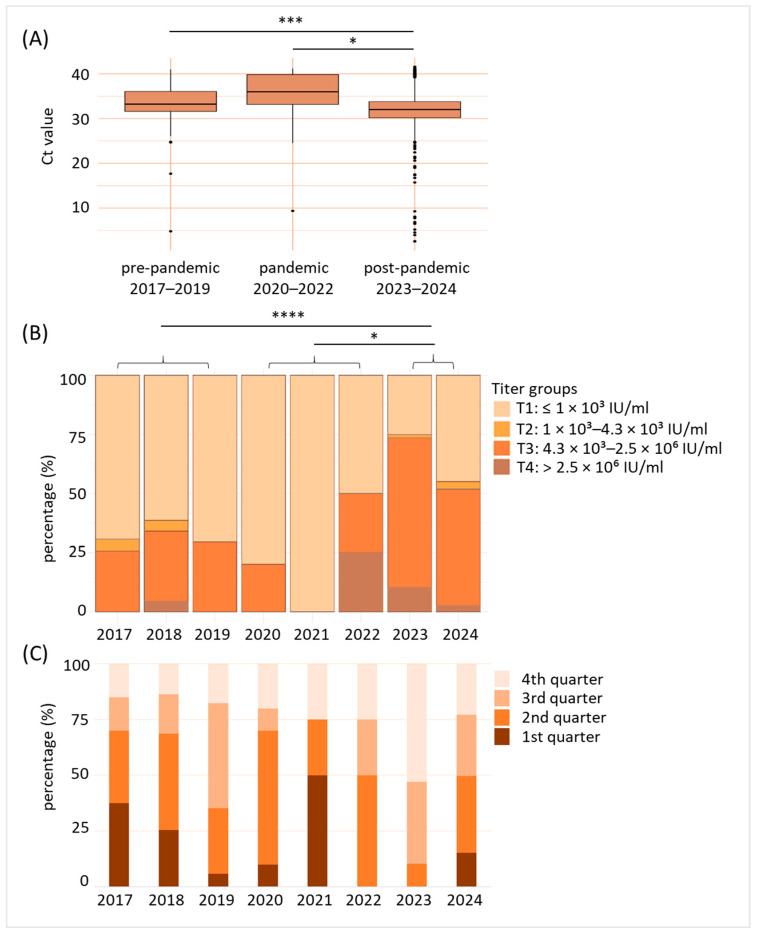
B19V viral titers were significantly enhanced after the SARS-CoV-2 pandemic. (**A**) Comparison of detected B19V cycle threshold (Ct)-values in blood donations of pre-pandemic (2017–2019), pandemic (2020–2022), and post-pandemic (2023–2024) years. * *p* < 0.05, *** *p* < 0.001. (**B**) Distribution of virus titer groups (T1–T4) of the individual years. T1: ≤1 × 10^3^ IU/mL, T2: 1 × 10^3^–4.3 × 10^3^ IU/mL, T3: 4.3 × 10^3^–2.5 × 10^6^ IU/mL, T4: >2.5 × 10^6^ IU/mL. Data shown are percentage (%) of B19V-positive blood donations for the year indicated belonging to the corresponding titer group. * *p* < 0.05, **** *p* < 0.0001. (**C**) Indication of B19V-positive blood donations by seasonal occurrence (categorization in 1st to 4th quarter of the year). Data shown are given in percentage (%).

As a next step, we analyzed the B19V prevalence before and after the SARS-CoV-2 pandemic in more detail, taking into account Ct-values and titers. We therefore defined comparable periods as pre-pandemic (2017–2019), pandemic (2020–2022) and post-pandemic (2023–2024). Our analysis revealed significantly decreased Ct-values in the post-pandemic phase when compared to both pre-pandemic (*p* < 0.001) and pandemic (*p* < 0.05) times (Figure 3A). As shown in Figure 3B, while during the pre-pandemic period and the first two years of the pandemic, up to 30% of B19V-positive donations revealed B19V titers within the third (4.3 × 10^3^–2.5 × 10^6^ IU/mL) or fourth (>2.5 × 10^6^ IU/mL) titer group, B19V virus titers substantially increased in 2022 (50% in titer group 3 or 4). A further increase was even observed in 2023 (>70% in titer group 3 or 4), before titers began to fall again in 2024 (>50% in titer group 3 or 4). As already shown for Ct values, pairwise comparisons revealed significant differences for B19V titers: Post-pandemic titers were significantly higher compared to pre-pandemic (*p* < 0.0001) and pandemic (*p* < 0.05) values. We also investigated in which part of the year the B19V cases occurred (Figure 3C). Our data reveal that in the pre-pandemic phase (2017–2019), the majority of B19V-positive cases occurred in the second and third quarter of a year. Up to 75% of the few B19V-positive donations observed during the pandemic period were found in the first and second quarters. In the post-pandemic year 2023, 90% of all cases were in the third or fourth quarter. In 2024, B19V-positive cases were detected throughout the year.

### 3.3. B19V Viremia Is Independent of Sex and ABO Blood Group, but Dependent on Age

**Figure 4:**
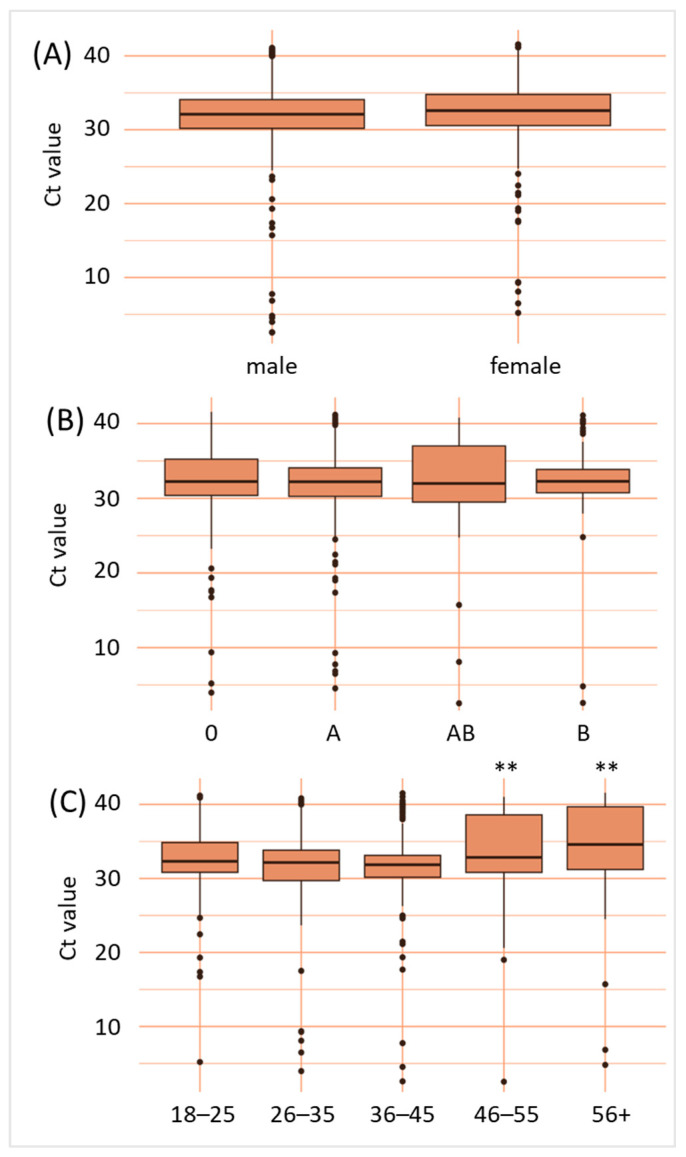
B19V viral loads are independent of sex and ABO blood group, but dependent on age. Data shown are B19V cycle threshold values according to sex (**A**), ABO blood group (**B**) and age given in years (**C**). ** *p* < 0.01.

Furthermore, we analyzed the influence of sex, ABO blood group, and age on the B19V viral load of all donations within the years 2017–2024. Regarding sex, 53% of the B19V-positive donors were male, while 47% were female, indicating a higher rate of B19V-positive females than within the donor population investigated (Table 2). However, when comparing Ct-values of male and female B19V-positive donors, our data did not reveal any significant difference (*p* > 0.05) (Figure 4A). ABO blood groups were distributed similarly to the general population (Table 2). However, the rate of B19V-positive blood donations in blood group A was slightly higher than expected. Again, no significant differences between the Ct-values of different ABO blood groups were detected (*p* > 0.05) (Figure 4B). As far as age groups are concerned, we found substantially higher numbers of younger blood donors being B19V-positive (Table 2): About 25% of B19V-positive donors were aged 18–25, 20% were between 26 and 35, and 27% were in the age group 36–45. Furthermore, 19% of B19V-positives were aged 46–55, and 9% were in the age group 56+. Together, the age group 18–45 make up 54% of the total blood donations and account for 72% of all B19V-positive blood donations, while the age group 45+ account for 46% of all blood donations and 28% of B19V-positive blood donations. Furthermore, a comparison of the Ct-values revealed that older age groups (46–55 and 56+) show significantly higher Ct-values, when compared to younger age groups (*p* < 0.01) (Figure 4C).

### 3.4. After 2 Years 39% of B19V-Positive Tested Blood Donors Are Still B19V-Positive

**Figure 5:**
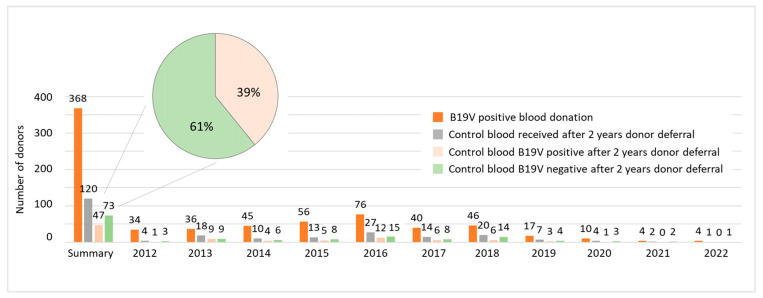
In total, 39% of B19V-positive blood donors are still tested positive for B19V DNA two years later. Data shown are the number of donors tested positive for B19V (orange), the number of donors, who obtained a control blood sample after the 2-year deferral (grey), the number of blood donors that were tested B19V-positive again (light orange) and those being tested negative (green) for each year as indicated. The summary section provides total numbers for the years 2012–2022, referring to all donors that were invited to provide a blood sample by the end of 2024.

At the Department for Transfusion Medicine in Salzburg, B19V-positive blood donors are deferred for 2 years before they are allowed to donate blood again. As B19V deferral modalities can be an issue for blood banks due to the shortage of suitable blood donors, we asked whether a shorter deferral would be more reasonable. Thus, we analyzed 368 B19V-positive blood donors who were invited to provide a control blood sample by the end of 2024. In total, 120 people returned for a follow-up blood test after the end of the donor deferral. Of these, 47 tested positive for B19V again and 73 tested negative. These results indicate that, after two years, 39% of B19V-positive blood donors still were tested positive for B19V DNA (Figure 5).

**Figure 6:**
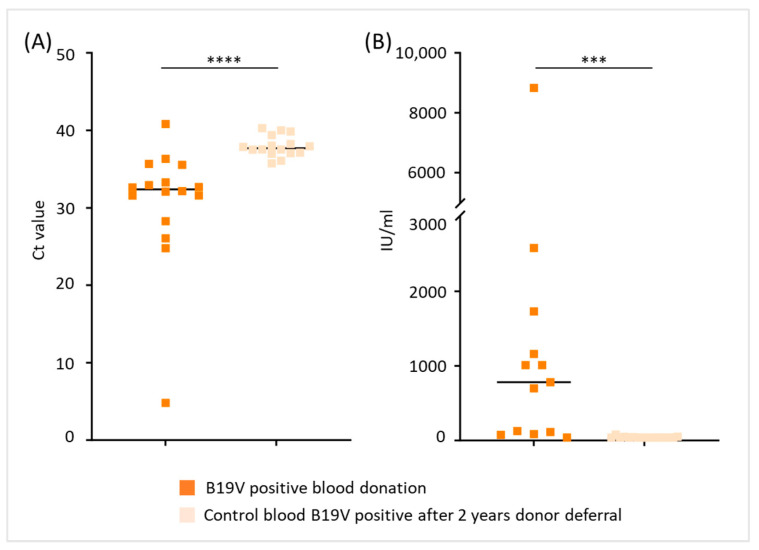
B19V load in blood donors tested positive again after a 2-year deferral period. (**A**) Ct values and (**B**) B19V titer values (IU/mL) of blood donors tested positive for B19V compared to Ct and titer values of their initial blood donation (*** *p* < 0.001, **** *p* < 0.0001). To obtain better descriptiveness of the figure shown in (**B**), data with >10,000 IU/mL of three individuals are not depicted in the plot.

With regard to B19V load in donors who tested positive again after a 2-year donor deferral period, Ct and titer values were available for the years 2017 to 2022 (*n* = 16). Donors retested positive had significantly higher Ct values (Figure 6A) and lower viral titers (<1 × 10^3^ IU/mL, Figure 6B) respectively, suggesting that B19V load declines over time.

## 4. Discussion

Our retrospective analysis shows a significant increase in parvovirus B19 prevalence among blood donors since the end of the SARS-CoV-2 pandemic in May 2023. Following years of steady and low prevalence (0.13% between 2012–2019), B19V rates strongly decreased in 2020–2021 (0.02%), coinciding with the implementation of widespread SARS-CoV-2 mitigation strategies such as lockdowns, mask-wearing and social distancing. Notably, we observed a sharp increase in B19V donor positivity starting in June 2023, simultaneously when SARS-CoV-2 mitigation strategies were lifted in Austria, which peaked in 2024 (2.7% in June 2024, 1.47% on average for 2024) and exceeded pre-pandemic levels by a more than 10 fold increase. This temporal pattern suggests that the disruption to viral transmission dynamics caused by the pandemic was a catalyst for the increase in B19V prevalence later on. Similar observations have been documented globally: Public health surveillance in early 2024 revealed unusually high parvovirus activity across 14 European countries [24]. In the United States, serological evidence of B19V infection rose from <3% during 2022–2023 to about 10% by June 2024 [25]. Concomitantly, the proportion of U.S. plasma donor samples exceeding high viremia thresholds (>1 × 10^4^ IU/mL) increased from 1.5% in late 2023 to 19.9% by 2024 [25]. Additionally, blood banks worldwide reported a strong increase in B19V prevalence among the donor populations [15,26,27,28]. Furthermore, a study in Austria reported no B19V cases in 2021 but a 35.5% positivity rate in 2023 among pregnant women [29]. In Israel, over 40% of all parvovirus infections recorded between 2015 and 2023 occurred in the year 2023 alone [30]. Taken together, these findings suggest a synchronous international increase in B19V incidence following the SARS-CoV-2 pandemic, indicating a shared underlying cause. This post-pandemic increase in B19V prevalence could be explained by a so-called “immunity gap” or “immunity debt” [31,32]. Parvovirus B19, like most other infectious pathogens, had significantly fewer transmission opportunities due to social distancing. Consequently and as already described for other viruses such as respiratory syncytial virus (RSV), seasonal influenza, parainfluenza, common coronaviruses and enterovirus [33,34,35,36], over a span of 1–2 years, a larger proportion of the population remained unexposed to infection. As pandemic restrictions eased, the virus was able to infect a larger pool of susceptible individuals, leading to a strong temporal increase in infections. It is important to note, that, also bacterial infections such as tuberculosis and pertussis significantly increased after the pandemic as well [37]. However, there could also be another explanation: SARS-CoV-2 may act as an inducer of latent virus reactivation. Indeed, there are several examples of viral infections having a transactivating function that allows another (latent) virus to reactivate. Human herpesvirus 6 (HHV-6) is known to reactivate the replicative cycle of Epstein–Barr virus (EBV), but was also suggested to interact with Cytomegalovirus (CMV), to activate human immunodeficiency virus (HIV)-1 and human papillomavirus (HPV) [38,39,40,41,42]. SARS-CoV-2 is suggested as viral transactivator, triggering reactivation different HHVs, CMV and EBV [43,44,45]. In addition, Lai et al., demonstrated in their study that immunosuppression and concurrent infections with SARS-CoV-2 are risk factors for B19V reactivation [46]. However, the timing of the B19V surge—right after pandemic measures were lifted—points toward an increased susceptibility of the population as the primary cause. Nevertheless, further research regarding host immune alterations post-COVID-19 is needed to fully exclude a compounding effect on B19V incidence.

Our data further revealed a significant decrease in Ct values in B19V-positive blood donations during the post-pandemic period (2023–2024), corresponding to higher viral titers when compared to both pandemic and pre-pandemic periods. Furthermore, we found B19V-positive donations all year around, whereas in pre-pandemic years B19V-positive cases occurred mainly in the 2nd and 3rd annual quarter (corresponding to spring and summer) of a year. The observation of atypical seasonality and high viremia, pointing towards a higher proportion of acute, primary B19V infections, is in line with other studies [15,25,30]. Notably, although blood-borne transmission of B19V is generally rare, higher viral loads indicate an increased risk of transmission to susceptible recipients [13,47,48].

Neither sex nor ABO blood group affected B19V titer values. Our demographic data show that younger blood donors (aged 18–45), who make up 54% of the total blood donor population, were more likely to test positive for B19V (72% of all cases) than the 45+ age group, who account for 46% of total blood donations and 28% of B19V-positive blood donations. Another study reported a substantial increase in B19V positivity among younger plasma donors in Europe, supporting our observation [49]. This finding aligns with the “immunity debt” hypothesis, as these younger blood donors would have had fewer opportunities for prior B19V exposure compared to older individuals. In addition to the “immunity debt”, these age groups are, due to care work, more likely in contact with children, for whom the sharpest increase in B19V prevalence was recently reported by the US Centers for Disease Control [25]. Children, being more likely to experience primary infections, would consequently present with higher viral loads and thus would infect their parents and older siblings more likely than the elderly population. Conversely, older donors with significantly higher Ct values (lower viral loads) might reflect a greater likelihood of pre-existing immunity from past infections.

The safest approach to prevent blood-borne B19V transmission is time-based deferral until the donor is no longer infectious. A key question is how long to defer a donor who is confirmed B19V-positive. Even though many blood collecting institutions worldwide do not screen routinely for B19V and the World Health Organization puts risk-based deferrals of blood donors in question instead [50], numerous blood banks screen blood donations routinely for B19V due to recipients safety reasons, applying different strategies when blood donors are screened positive for B19V. While the Joint UK Blood Transfusion Services Professional Advisory Committee (JPAC) recommends deferring donors with confirmed parvovirus B19 infection until at least 4 weeks after full recovery from symptoms (https://www.transfusionguidelines.org/dsg/wb/guidelines/parvovirus-b19#:~:text=Discretionary, last accessed on 17 June 2025), in the Netherlands and Italy B19V-positive donors are deferred for 6 months [3,51]. In the US, there are no guidelines so far, but usually donors with symptoms of B19V disease are deferred to ensure clearance of B19V DNA [52]. In Germany, a positive result for Parvovirus B19 (B19V) in blood donation screening typically leads to a deferral for up to 12 months [28], but the deferral period may vary and is determined by the local blood donation service. Our data revealed that 39% of B19V-positive blood donors providing a follow-up sample after the currently applied 2-year deferral approach in Salzburg were still positive for B19V DNA. However, we are aware that the two-year deferral is a relatively strict approach, putting a high organizational burden on the blood donation system and demands above-average donation frequency of blood donors. As B19V DNA may persist in blood and other tissues for extended periods [8,9,10], we implemented the 2-year deferral period for donors positive for B19V DNA. However, a crucial consideration is whether persistent DNA indeed represents infectious virus or merely residual, non-infectious viral fragments. The qPCR method used in the present study does not differentiate between infectious virions and non-viable genetic material. Therefore, cases of long-term DNA persistence, especially if associated with low viral loads, may not pose a significant transfusion transmission risk on recipients. Even though the 2-year deferral might be a strict approach, putatively leading to the loss of committed blood donors, to date it cannot be excluded that these 39% persistently positive donors harbor infectious viruses. Therefore, the 2-year deferral is an effective strategy to protect high-risk transfusion recipients.

Our present study possesses several strengths such as the large sample size, encompassing 441,084 blood donations screened over a 12-year period, providing a robust dataset for epidemiological analysis. The data collection, including prevalence rates, Ct values, viral titer, seasonal distribution patterns, and donor demographics, allows for a detailed examination of B19V dynamics. In addition, the inclusion of follow-up data on B19V DNA persistence in a cohort of initially positive donors provides valuable information regarding donor management policies. However, as a single-center study, our findings, while consistent with international observations, may not be generalizable to other populations, as local donor demographics and other environmental factors could play a role. Another limitation is that B19V genotyping was not performed, which could provide additional insights into whether specific viral variants were associated with the observed post-pandemic increase.

## 5. Conclusions

This long-term, retrospective analysis of Austrian blood donors revealed a substantial rise in parvovirus B19 prevalence and viral loads after the SARS-CoV-2 pandemic. Our observations are consistent with international reports and are likely due to an ‘immunity debt’ accrued during years of pandemic-related public health interventions. These results suggest that younger donors were particularly affected by the post-pandemic increase in B19V infections. Additionally, we detected higher viral titers compared to the pandemic and pre-pandemic periods, suggesting an increased potential risk to transfusion recipients. Of particular interest is the persistence of B19V DNA in a substantial proportion of donors, with 39% remaining positive after a two-year deferral period. Strict deferral strategies and targeted B19V screening may be necessary, particularly during outbreak periods, to protect high-risk transfusion recipients.

## Figures and Tables

**Table 1:** Cohort characteristics of blood donors in Salzburg, Austria, 2012–2024 and during the pre-pandemic (2017–2019), pandemic (2020–2022) and post-pandemic (2023–2024) phases. Data are shown as total numbers or percentages as indicated.

	Blood Donations 2012–2024	Pre-Pandemic 2017–2019	Pandemic 2020–2022	Post-Pandemic 2023–2024
Total number of donations	441,084	100,936	101,037	65,822
Sex (in %)				
Male	59.93	59.50	56.95	57.74
Female	40.07	40.50	43.05	42.26
ABO blood group (in %)				
A	38.52	38.92	38.16	38.11
B	10.68	10.53	10.67	10.76
AB	4.32	4.35	4.26	4.33
0	46.48	46.20	46.91	46.80
Age groups (age at the time of donation in years, given in %)				
18–25	18.21	19.38	17.72	15.13
26–35	18.05	17.53	18.99	18.15
36–45	18.53	17.85	18.09	19.52
46–55	24.12	25.42	23.17	21.46
56–70	21.08	19.83	22.03	25.75

**Table 2:** Cohort characteristics of blood donations and B19V-positive blood donations in Salzburg, Austria, 2017–2024.

	Blood Donations 2017–2024	B19V-Positive Blood Donations 2017–2024
Total number of donations	267,795	674
Sex (in %)		
Male	58.06	53.45
Female	41.94	46.55
ABO blood groups (in %)		
A	38.40	47.14
B	10.65	7.41
AB	4.31	2.13
0	46.64	43.32
Age groups (age at the time of donation in years, given in %)		
18–25	17.41	25.19
26–35	18.22	19.88
36–45	18.48	26.97
46–55	23.35	19.09
56–70	22.54	8.87

## Data Availability

The data presented in this study are available on request from the corresponding author due to donor privacy.

## Abbreviations

The following abbreviations are used in this manuscript:

COVID-19	Coronavirus disease 2019
Ct	Cycle threshold
DNA	Deoxyribonucleic Acid
EDTA	Ethylenediaminetetraacetic acid
HAV	Hepatitis-A Virus
HBV	Hepatitis-B Virus
HCV	Hepatitis-C Virus
HIV	Human Immunodeficiency Virus
JPAC	Joint UK Blood Transfusion Services Professional Advisory Committee
NAT	Nucleic Acid Amplification Technology
B19V	Parvovirus B19
(q)PCR	(quantitative) polymerase chain reaction
SARS-CoV-2	Severe acute respiratory syndrome coronavirus 2
WHO	World Health Organization
WNV	West Nile Virus
